# Supplementary material for: Transcriptome for the breast muscle of Jinghai yellow chicken at early growth stages
Source: PeerJ. 2020 Apr 15;8:e8950. doi: 10.7717/peerj.8950 (PMC7166044; doi:10.7717/peerj.8950)
Supplement: Table S1 [file peerj-08-8950-s001.docx]

**Table S1 The sequences of primers for β-actin and DEGs verified by qPCR in the study**

| **Name of genes** | **Primer sequence (5’-3’)** | **Product length (bp)** | **Annealing temperature (°C)** |
| --- | --- | --- | --- |
| MYH1F-F | AGAAGCAGCGTGAAGAGC | 75 | 60°C |
| MYH1F-R | TGAGACCCATCAGGTAAGC |  |  |
| ACTC1-F | CAGAAGCACCCTTGAACC | 203 | 60°C |
| ACTC1-R | ATGAGGCAAAGCATAACC |  |  |
| C1QC-F | GCTGAAGGGAGCCAAAGG | 212 | 60°C |
| C1QC-R | AGAACGCTGACTGGTGCA |  |  |
| ANXA1-F | CAGATCAAAGCTGCCTAT | 123 | 60°C |
| ANXA1-R | ATCAAACTGAGCTGGAGT |  |  |
| PLPPR4-F | AAAGTCATTCCCATCTCAAC | 204 | 60°C |
| PLPPR4-R | AGAAAGCCACAGTAAACATC |  |  |
| RACGAP1-F | CCATAGTGGCAAAGACGA | 164 | 60°C |
| RACGAP1-R | GGATTCCACCTGCTTAGAG |  |  |
| β-actin-F | CAGCCATCTTTCTTGGGTAT | 169 | 60°C |
| β-actin-R | CTGTGATCTCCTTCTGCATCC |  |  |
