# Supplementary material for: Transcriptome for the breast muscle of Jinghai yellow chicken at early growth stages
Source: PeerJ. 2020 Apr 15;8:e8950. doi: 10.7717/peerj.8950 (PMC7166044; doi:10.7717/peerj.8950)
Supplement: Table S2 [file peerj-08-8950-s002.docx]

**Table S2 The results of quality control for the clean data from RNA-seq of each sample**

| **Sample name** | **Raw reads** | **Clean reads** | **Clean bases** | **Q-sorce 20 (%)** | **Q-sorce 30 (%)** | **GC content (%)** |
| --- | --- | --- | --- | --- | --- | --- |
| F4F_1 | 57332426 | 53990618 | 8.1G | 95.55 | 90.24 | 54.58 |
| F4F_2 | 65164376 | 61663414 | 9.25G | 95.63 | 90.36 | 54.13 |
| F4F_3 | 51857508 | 49118588 | 7.37G | 96.58 | 92.14 | 54.51 |
| F8F_1 | 62961680 | 59233086 | 8.88G | 96.66 | 92.22 | 54.19 |
| F8F_2 | 52949754 | 50073278 | 7.51G | 96.78 | 92.52 | 54.58 |
| F8F_3 | 54514850 | 51990782 | 7.8G | 96.91 | 92.69 | 52.85 |
| F4S_1 | 53045954 | 50337158 | 7.55G | 96.46 | 91.93 | 54.68 |
| F4S_2 | 56550890 | 53588300 | 8.04G | 96.49 | 92.03 | 54.43 |
| F4S_3 | 59058956 | 56081520 | 8.41G | 96.45 | 91.88 | 53.89 |
| F8S_1 | 52731880 | 49998426 | 7.5G | 96.79 | 92.51 | 54.28 |
| F8S_2 | 52558780 | 49491048 | 7.42G | 96.82 | 92.55 | 54.4 |
| F8S_3 | 65234778 | 60733042 | 9.11G | 96.55 | 92.1 | 53.89 |
| M4S_1 | 52827100 | 50822036 | 7.62G | 97.28 | 93.27 | 50.82 |
| M4S_2 | 54511420 | 51581852 | 7.74G | 96.56 | 92.07 | 53.38 |
| M4S_3 | 50497564 | 47770614 | 7.17G | 96.55 | 92.13 | 54.8 |
| M8S_1 | 52367686 | 49864380 | 7.48G | 96.58 | 92.12 | 54.2 |
| M8S_2 | 56144980 | 53234338 | 7.99G | 96.41 | 91.8 | 54.21 |
| M8S_3 | 51435032 | 48911326 | 7.34G | 96.54 | 92.06 | 53.52 |
