# Supplementary material for: Transcriptome for the breast muscle of Jinghai yellow chicken at early growth stages
Source: PeerJ. 2020 Apr 15;8:e8950. doi: 10.7717/peerj.8950 (PMC7166044; doi:10.7717/peerj.8950)
Supplement: Table S3 [file peerj-08-8950-s003.docx]

**Table S3 The comparison result between the reference genome and high-quality clean reads of each sample**

| **Sample name** | **F4F_1** | **F4F_2** | **F4F_3** | **F8F_1** | **F8F_2** | **F8F_3** | **F4S_1** | **F4S_2** | **F4S_3** |
| --- | --- | --- | --- | --- | --- | --- | --- | --- | --- |
| **Total clean reads** | 53990618 | 61663414 | 49118588 | 59233086 | 50073278 | 51990782 | 50337158 | 53588300 | 56081520 |
| **Total mapped reads** | 39979073 (74.05%) | 46433575 (75.3%) | 36903306 (75.13%) | 45192892 (76.3%) | 37882378 (75.65%) | 41455326 (79.74%) | 37382444 (74.26%) | 40139684 (74.9%) | 42759464 (76.25%) |
| **Multiple mapped** | 2360229 (4.37%) | 2660976 (4.32%) | 2118830 (4.31%) | 2501099 (4.22%) | 2182294 (4.36%) | 2033510 (3.91%) | 1945431 (3.86%) | 2295898 (4.28%) | 2431713 (4.34%) |
| **Uniquely mapped** | 37618844 (69.68%) | 43772599 (70.99%) | 34784476 (70.82%) | 42691793 (72.07%) | 35700084 (71.3%) | 39421816 (75.82%) | 35437013 (70.4%) | 37843786 (70.62%) | 40327751 (71.91%) |
| **Reads map to '+'** | 18764992 (34.76%) | 21845413 (35.43%) | 17346135 (35.31%) | 21282361 (35.93%) | 17781505 (35.51%) | 19697119 (37.89%) | 17667390 (35.1%) | 18894756 (35.26%) | 20127743 (35.89%) |
| **Reads map to '-'** | 18853852 (34.92%) | 21927186 (35.56%) | 17438341 (35.5%) | 21409432 (36.14%) | 17918579 (35.78%) | 19724697 (37.94%) | 17769623 (35.3%) | 18949030 (35.36%) | 20200008 (36.02%) |
| **Non-splice reads** | 17753015 (32.88%) | 21358888 (34.64%) | 16690180 (33.98%) | 20332084 (34.33%) | 16886410 (33.72%) | 19473028 (37.45%) | 17067568 (33.91%) | 18249298 (34.05%) | 19428640 (34.64%) |
| **Splice reads** | 19865829 (36.79%) | 22413711 (36.35%) | 18094296 (36.84%) | 22359709 (37.75%) | 18813674 (37.57%) | 19948788 (38.37%) | 18369445 (36.49%) | 19594488 (36.56%) | 20899111 (37.27%) |

NOTE: (1) Total mapped reads: The number of sequences located on the genome; (2) Multiple mapped: The number of sequences with multiple alignment sites on the reference genome; (3) Uniquely mapped: The number of sequences with unique alignment positions on the reference genome. (4) Reads map to '+'，Reads map to '-': The number of sequences compared to positive and negative chains in the genome; (5) Splice reads: The number of sequences aligned to two exons; (6)Non-splice reads: The number of sequences aligned to one exon.
