# Supplementary material for: Transcriptome for the breast muscle of Jinghai yellow chicken at early growth stages
Source: PeerJ. 2020 Apr 15;8:e8950. doi: 10.7717/peerj.8950 (PMC7166044; doi:10.7717/peerj.8950)
Supplement: Table S4 [file peerj-08-8950-s004.docx]

**Table S4 The body weight at 4 and 8 weeks of Jinghai yellow chickens used in the study**

| **Age of four weeks** | **Weight(g)** | **Age of eight weeks** | **Weight(g)** |
| --- | --- | --- | --- |
| F4F_1 | 300 | F8F_1 | 750 |
| F4F_2 | 310 | F8F_2 | 755 |
| F4F_3 | 310 | F8F_3 | 780 |
| F4S_1 | 225 | F8S_1 | 540 |
| F4S_2 | 205 | F8S_2 | 505 |
| F4S_3 | 180 | F8S_3 | 575 |
| M4S_1 | 215 | M8S_1 | 565 |
| M4S_2 | 175 | M8S_2 | 615 |
| M4S_3 | 240 | M8S_3 | 535 |

**NOTE:** F4F or F8F：female, 4 weeks or 8 weeks, fast-growing; F4S or F8S：female, 4 weeks or 8 weeks, slow-growing; M4F or M8F: male, 4 weeks or 8 weeks, fast-growing
